# Habitual snoring and atopic state: correlations with respiratory function and teeth occlusion

**DOI:** 10.1186/1471-2431-12-175

**Published:** 2012-11-07

**Authors:** Anna Maria Zicari, Giuseppe Marzo, Anna Rugiano, Camilla Celani, Maria Palma Carbone, Simona Tecco, Marzia Duse

**Affiliations:** 1 Department of Paediatric Science, University La Sapienza, Rome, Italy; 2 Department of Life, Health and Enviromnental Science, University of L’Aquila, L’Aquila, Italy

**Keywords:** Sleep-disordered breathing (SDB), Atopy, Dento-facial morphology, Allergy, Oral breathing, Snoring

## Abstract

**Background:**

Allergy represents a risk factor at the base of sleep-disordered breathing in pediatric age. Among allergic diseases, the atopy is characterized by a tendency to be “hyperallergic.” Sleep-disordered breathing is also known in orthodontics as correlated with the morphology of craniofacial complex. The aim of this study was to investigate the relation between atopy and sleep-disordered breathing (oral breathers with habitual snoring), comparing atopic children with sleep-disordered breathing (test group) with nonatopic ones with sleep-disordered breathing (control group), in the prevalence of dento-skeletal alterations and other risk factors that trigger sleep-disordered breathing, such as adenotonsillar hypertrophy, turbinate hypertrophy, obesity, and alteration of oxygen arterial saturation.

**Methods:**

In a group of 110 subjects with sleep-disordered breathing (6 to 12 years old), we grouped the subjects into atopic (test group, 60 subjects) and nonatopic (control group, 50 subjects) children and compared the data on the following: skin allergic tests, rhinoscopy, rhinomanometry, night home pulsoxymetry, body mass index, and dento-facial alterations.

**Results:**

Even if our results suggest that atopy is not a direct risk factor for sleep-disordered breathing, the importance of a physiologic nasal respiration in the pathogenesis of sleep-disordered breathing seems to be demonstrated in our study by the higher prevalence of hypertrophy in the adenotonsillar lymphatic tissue, odontostomatological alterations, alterations of the oxygen saturation to pulsoxymetry, and higher prevalence of obesity observed in our children with sleep-disordered breathing, in percentages higher than that of the general pediatric population previously observed in the literature.

**Conclusions:**

The importance of a physiologic nasal respiration in the pathogenesis of sleep-disordered breathing is demonstrated in our study.

## Background

Sleep-disordered breathing (SDB) in pediatric age represents a group of disorders that occur or intensify during sleep. They include a spectrum of hypoventilatory obstructive disorders:

The primary snoring or habitual snoring without alterations of the sleeping pattern and gaseous exchanges;

The upper airway resistance syndrome with fragmentation of the sleep and alteration of gaseous exchanges;

The obstructive sleep apnoea (OSA) with alterations of the sleeping pattern and desaturating events [1-3].

Despite the scarce attention to the problem, because many hypoventilatory obstructive disorders are not always pointed out by the parents spontaneously, these have been representing in the last 10 years, a worrisome cause of morbidity in pediatric age, with significant impact on the socioeconomic aspect and the quality of life of children and their families.

By simply investigating one of the most frequent symptoms, habitual snoring, we learn from the anamnestic studies of the recent literature, that its prevalence varies from 2.5 to 34.5% [4].

The large range is mainly related to the lack of standardization of the definitions used by the single authors: in more “restrictive” studies, habitual snoring is pointed out only if the symptom is “always” present; therefore, the prevalence varies from 1.5 to 6%; if the symptom instead falls under the general definition of “habitual” (without frequency ratio), the prevalence increases from 5 to 12% [5].

Within the sphere of multidisciplines, allergy represents a risk factor at the base of this phenomenon, which is often attributed as the cause of SDB [6,7]. Indeed, the allergic inflammation concurs directly to the mechanical obstruction of the nasal cavities (turbinate hypertrophy) and/or nasopharynx (adenotonsillar hypertrophy) and indirectly to odontostomatological alterations; however, to date, there is a debate on how and to what extent allergy is related with the respiratory disorders.

Among allergic diseases, the atopy elevated levels of total and allergen-specific IgE in the serum of patient, leading to positive skin-prick tests to common allergens [8]. A patient with atopy typically presents with one or more of the following: eczema (atopic dermatitis), allergic rhinitis (hay fever), allergic conjunctivitis, or allergic asthma [8].

Within the sphere of multi-disciplines anyhow, SDB is known in orthodontics as correlated with the morphology of craniofacial complex [9].

Today, in literature, it still lacks evidence of a particular rule associated with the atopy in relation to SDB.

The development of craniofacial structures and nasal breathing has long generated interest and controversy.

This controversy lies in whether nasal obstruction leads to oral breathing or not and, especially, in whether it is capable of causing changes in dentofacial development. There has always been a certain belief that nasal obstruction affects facial growth. However, there is no conclusive evidence that there is a causal link between both factors. Normal breathing requires the free flow of air through the nose. Respiratory function associated with chewing and swallowing, plus correct muscular action of the lips and tongue, encourages adequate facial development and growth. Upper airway obstruction involving oral breathing can be harmful when it is present at the time of development of the face, orofacial skeleton, and teeth [10].

In our study on a sample of children with habitual snoring and oral breathing, we wanted to evaluate atopy because this may affect some risk factors that trigger the SDB, such as adenotonsillar hypertrophy, turbinate hypertrophy, obesity, impaired arterial oxygen saturation, and dento-skeletal abnormalities.

The identification of diseases at the base of the upper airway obstruction, which atopy is not solely responsible, is essential to set a correct and timely multidisciplinary therapeutic approach to prevent chronic disorders and the onset of complications that are sometimes permanent.

In fact, the atopy could adversely affect the clinical condition of the young patients, causing a chronic inflammatory state that is in addition to that caused by upper respiratory tract infections.

Several studies [11-13] have highlighted the strong association between atopy and the severity of sleep disorders, of which, atopy is considered an important risk factor that requires a greater attention in the setting of the correct treatment plan.

## Methods

Among the children coming to the Department of the Special Service of Immunology and Pediatric Allergology of “Sapienza” University in Rome, we selected a group of habitual snorers (110 subjects), through a standardized questionnaire for SDB, [14] 66 of which are male subjects (60%) and aged between 6 and 12 years (average 8.2 ± 1.5) (Table 1). Subjects who had previously underwent orthodontic treatments or those with facial-cranial malformations, genetic syndromes, and neuromuscular pathologies were excluded.

**Table 1:** Description of the sample

	**Atopic SDB subjects**	**Non atopic SDB subjects**	**MEAN AGE ( SD)**
	**(60 subjects)**	**(50 subjects)**	**Range 6–12 years**
**Males**	36/60	30/50	8.5 ± 1.5
**Females**	24/60	20/50	8.3 ± 1.6

The ethical approval for the experimental research was given by the ethics committee of the university. The research was carried in compliance with the Helsinki Declaration. Informed consent has been obtained from the parents of the children involved in the study.

The subjects were then divided into two groups on the basis of the skin allergic tests (Lofarma allergens skin prick test) for common inhaled allergens (Dermatophagoides pteronyssinus, Dermatophagoides farinae, Alternaria tenuis, Parietaria officinalis, Cynodon dactylon, Olea europea, Lolium, cat and dog dander).

Two groups were individuated: (i) subjects with SDB and atopy (test group, 60 subjects), and (ii) subjects with SDB without atopy (control group, 50 subjects).

All the selected subjects performed the following:

Clinical evaluation of the degree of tonsillar hypertrophy, according to the criteria of Mallampati. Patients were classified as follows: (i) patients with a low Mallampati scores (scores 1 and 2), and (ii) patients with high Mallampati scores (scores 3 and 4) [15];

Anterior rhinoscopy.

Assessment of adenoid hypertrophy through fibroendoscopia, considering as hypertrophic adenoid grades 3 and 4 according to the criteria of Cassano [16].

Active anterior rhinomanometry (Rhinospir PRO 164) before and after nasal decongestion test. According to the rules set forth by the International Standardization Committee on Objective Assessment of Nasal Airway, corrected for the pediatric reference values indicated in the literature. [17] The rhinomanometry was considered positive when the degree of obstruction ranged from moderate (43-56% of p.v.) to very severe (<29% of p.v.).

Night home pulsoxymetry (Respironics 920M, Profox software): with desaturation clusters of 5 or more between 10 and 30 minutes; the test was defined “**positive**” if 3 or more desaturation clusters were present, and three desaturations with drop of SaO_2_ below 90%; “**negative**” in the absence of desaturation clusters and drops of SaO_2_ below 90%; and “**nonconclusive**” if less than 3 desaturation clusters were present [18].

Body mass index (BMI) evaluation was subdivided into four categories: underweight BMI < 3rd percentile, normal weight between 3rd and 90th percentile; overweight between 90th and 97th percentile; obese > 97th percentile [19].

Lateral skull radiograph with orthopantomography of the dental arches on which we performed cephalometric analysis according to the Tweed method to assess skeletal class.

Objective intraoral examination with evaluation of the dental class, overbite, overjet, midlines, cross-bite, and bad habits (atypical deglutition, labial incompetence, and suction of the finger and inner lip).

Alginate moulds of the dental arches and study of the plaster moulds with evaluation of the dental arch perimeter, the transpalatal width, and analysis of the space.

### Statistical analysis

The two groups considered in this case control study were as follows: (i) the test group that included SDB atopic children, and (ii) the control group that included SDB nonatopic children.

The χ2 test was used to compare proportions between the test and control groups for all the considered variables. The statistic meaning was defined at p < 0.05 level.

## Results

By comparing SDB atopic subjects (60/110) (test group) vs. SDB nonatopic subjects (50/110) (control group), the prevalence of the parameters taken in consideration showed the following:

Adenoid hypertrophy: 36% (21/60) in the test group vs. 78% (39/50) in the control group (**p** = **0**.**001**) (Table 2).

**Table 2:** Main results in the sample

	**Atopic SDB subjects**	**Non atopic SDB subjects**	**Statistical significance**
	**(60 subjects)**	**(50 subjects)**	
**Adenoid Hypertrophy**	36% (21/60)	46% (39/50)	P=0.001
**Tonsillar hypertrophy**	32% (19/60)	46% (23/50)	n.s.
**Turbinate hypertrophy**	68% (41/60)	60% (30/50)	n.s.
**Overweight**	16.6% (10/60)	58% (29/50)	P=0.005
**Positive rhinomanometry (obstruction from 43% to <29% of p.v.**	80% (48/60)	78% (39/50)	n.s.
**Positive/Non conclusive pulsoxymetry**	50% (30/60)	42% (21/50)	n.s.

n.s.: not significant.

Tonsillar hypertrophy: 32% (19/60) in the test group vs. 46% (23/50) in the control group (not significant difference) (Table 2).

Turbinate hypertrophy: 68% (41/60) in the test group vs. 60% (30/50) in the control group (not significant difference) (Table 2).

Over-weight: 16.6% (10/60) in the test group vs. 58% (29/50) in the control group (**p** = **0**.**005**) (Table 2).

Positive rhinomanometry (obstruction from 43% to <29% of p.v.): 80% (48/60) in the test group vs. 78% (39/50) in the control group (not significant difference) (Table 2).

Positive/nonconclusive pulsoxymetry: 50% (30/60) in the test group vs. 42% (21/50) in the control group (not significant difference) (Table 2).

Decrease of the transversal palatal diameter: 65% (39/60) in the test group vs. 88% (44/50) in the control group (**p** = **0**.**04**, significant difference) (Table 3).

**Table 3:** Relationship between orthodontic anomalies and atopy

	**Reduced transversal palatal diameter N (%)**	**Skeletal class II**	**Cross-byte**	**Atipic swallowing**
		**N (%)**	**N (%)**	**N (%)**
**Atopic SDB subjects**	39/60 (65%)	24/60 (40%)	21/60 (35%)	54/60 ( 90%)
**Non atopic SDB subjects**	44/50 (88%)	24/50 (48%)	15/50 (30%)	47/50 (94%)
p	P<0.05 (*)	n.s.	n.s.	n.s.

Ns: not significant.

Second skeletal class: 40% (24/60) in the test group vs. 48% (24/50) in the control group (not significant difference) (Table 3).

Cross-bite: 35% (21/60) in the test group vs. 30% (15/50) in the control group (not significant difference) (Table 3).

Atypical deglutition: 90% (54/60) in the test group vs. 94% (47/50) in the control group (not significant difference) (Table 3).

## Discussion

From epidemiologic studies on multiple risk factors able to affect respiratory disorders during sleep in the pediatric age, various predisposing conditions may coexist in the same subject, thus requiring a multidisciplinary approach.

Recent evidence has suggested that prematurity, maternal smoking during pregnancy, maternal age and weight gain during pregnancy, prenatal complications (such as maternal high blood pressure and gestational diabetes), and perinatal complications related to prematurity are associated with childhood SDB [20]. Also, low socioeconomic status and race seem to influence the incidence of prenatal and perinatal complications [20]. A recent study also indicates familial clustering of SDB (SDB caused by adenotonsillar or tonsillar hypertrophy), which is an important information for clinicians [21].

Our study, performed on SDB children, aimed at estimating the prevalence of the main risk factors with the analysis of the role that the atopy has in determining and/or favoring them, performing a comparison between SDB atopic children (test group) and SDB nonatopic children (control group).

In this sample of SDB children, a prevalence of atopy (55%) higher than that observed in previous literature regarding the general pediatric Caucasian population (10-15%), as reported in other studies, [22] was observed probably because of a selection bias that was expected because the patients were consulting a Special Service of Pediatric Allergology. This observation does not alter the validity of the results because the aim of this study is to highlight the differences between 60 SDB atopic children and 50 SDB nonatopic children.

A high prevalence of hypertrophy in the adenotonsillar lymphatic tissue in the whole sample of SDB children (54.5% adenoid hypertrophy, 38.1% tonsillar hypertrophy, and 21.5% tonsillar and adenoid hypertrophy) was also observed. This prevalence resulted significantly higher in SDB nonatopic subjects (control group) with respect to SDB atopic subjects (test group), suggesting that in our cases, the infective etiology seems to prevail, in contrast with some works [23] that attribute an immunogenic role in the adenoid hypertrophy, [24] to the allergic reaction. Recent literature shows that pediatric SDB patients with adenoid hypertrophy could be effectively treated with 4-week course of mometasone furoate, whereas allergy, obesity, and sinusitis seem not to affect the result of the treatment [25].

With recent studies as basis, adenotonsillar enlargement and tonsillar hypertrophy seem to mediate at least in part the epidemiologic association observed between SDB (mostly sleep apnea) and wheezing/asthma [26]. Elevated concentrations of leukotrienes and oxidative stress markers have been detected in the exhaled breath condensate of children with asthma and probably contribute to bronchoconstriction [26]. Moreover, SDB children with sleep apnea have increased expression of leukotrienes and leukotriene receptors in adenotonsillar tissue [26]. Probably, viral respiratory infections may induce inflammation and oxidative stress in the asthmatic airway, enhancing not only the bronchospasm but also the biosynthesis of leukotrienes within the pharyngeal lymphoid tissues, which promote adenotonsillar enlargement and sleep apnea [26].

In the whole sample of SDB children, modest prevalence of obesity was observed, higher than that of the general pediatric population previously observed in the literature (35.4% in our SDB sample vs. 11%), [27] which is mainly prevalent (at a statistical significant level) in the control group of SDB nonatopic subjects (58% in the control group and 16.6% in the test group): contrary to what is observed in adult age, [28] the role of obesity in determining SDB is still debated in pediatric age [29].

In addition, high prevalence of nasal obstruction to rhinomanometry and alterations of the oxygen saturation to pulsoxymetry in the whole sample of SDB children (79% and 46.3%, respectively in our whole sample) were detected, which seem not related in significantly statistical terms, with the atopy. Despite the said exams being not resolutive for SDB diagnosis, they appear to be useful to point out and follow up possible complications associated with nasal obstruction, oral respiration, and allergic inflammation. In a recent study, pediatric patients with positive environmental allergic rhinitis showed SDB and sleepiness scores (on questionnaires) higher than population with normal values, suggesting that children with allergic rhinitis are at increased risk for SDB, and screening should be considered in this population [30].

Generally, rhinomanometric values are in agreement with symptom severity and duration (persistent and intermittent allergic rhinitis) and the degree of allergic inflammation (concentration of inflammatory cytokines) [31] in patients affected by allergic rhinitis. Our results suggest that the nasal obstruction, despite the atopic or nonatopic state of the subjects, appears correlated to rhinomanometry alterations.

The presence of numerous odontostomatological alterations, with various percentages in the whole sample, was observed: (a) significant reduction of the transverse diameter, equal to 75% of the cases in the whole sample; (b) a second skeletal class in 43.6% of the cases in the whole sample; (c) a cross-bite in 32.7% of the cases in the whole sample; and (d) an atypical deglutition in 91% of the cases in the whole sample.

About the palatal diameter, literature reports an increased prevalence of SDB in the population with cleft palate, with an even greater prevalence in patients with Pierre Robin syndrome [32].

The presence of numerous odontostomatological alterations in our sample of SDB children, both atopic or nonatopic, suggests that nasal obstruction, no matter how it is induced, is associated with altered dentofacial development at the point that orthodontic therapy has been proposed as solution for respiratory problems and oral breathing [33,34]. With the data in literature as basis, craniofacial features considered to be risk factors for SDB include small mandible and/or high and narrow hard palate associated with a narrow nasomaxillary complex [35]. At the same time, the degree of nasal obstruction is correlated to the severity of snoring and OSAS so that the use of rhinomanometry is suggested as screening test before polysomnigraphic recording in children with suspected OSAS [36].

In particular, the high percentage of children with atypical deglutition detected in our whole sample, reflects the literature data that show how this disorder is present in 50% of three-year-old children and persists till twelve years of age, in 25% of the cases [22-39]. In any case, regardless of the atopic or nonatopic state, the atypical deglutition seems to be significantly related to the oral breathing because of the high prevalence of this disorder found in our whole sample of SDB children. Recently, a research stated that a cranial lateral cephalometry is required to study cephalometric characteristics if polysomnography points out any pathology referring to SDB [40].

Considering the two groups of atopic and nonatopic children, we observed some little differences between the two groups, although not statistically significant.

These odontostomatological characteristics were found in slightly higher percentages in the group of SDB nonatopic subjects (control group), which coincides with the group with greater adenoid hypertrophy. The slightly higher prevalence of decrease of trasversal palatal diameter in nonatopic group seems related to the oral breathing because of adenoid hypertrophy that is prevalent in this group. Cross-bite resulted with a slightly higher prevalence in SDB atopic subjects compared with nonatopic group, although no significance was detected (35% vs. 30%), in accordance with a previous study, [38] suggesting a possible genetic relation.

Considering the role of atopy in SDB subjects, on the basis of our observations, atopy seems not often related in significantly statistic terms with various risk factors that trigger SDB (adenotonsillar hypertrophy, turbinate hypertrophy, odontostomatological alterations) and with particular dento-facial characteristics, except for a reduced transversal palatal diameter (p < 0.05). Children with SDB also show a high prevalence of atypical deglutition that certainly contributes to trigger malocclusions because of chronic oral respiration.

The limitation of this study concerns the selection of subjects that was made not in a pediatric general population but in a sample of children who have been evaluated in a service of pediatric allergology (in which it is obvious to find a high prevalence of atopic children).

## Conclusions

In conclusion, the key point of the characteristics of SDB children seems to be the nasal obstruction, and even if our results suggest that atopy is not a direct or important risk factor for snoring and OSAS, the importance of a physiologic nasal respiration in the pathogenesis of SDB seems to be demonstrated in our study by the higher prevalence of the following: (i) hypertrophy in the adenotonsillar lymphatic tissue, (ii) numerous odontostomatological alterations, (iii) nasal obstruction to rhinomanometry and alterations of the oxygen saturation to pulsoxymetry, and (iv) modest prevalence of obesity—characteristics observed in our whole sample of SDB children, in percentages higher than that of the general pediatric population previously observed in the literature.

## Competing interests

The authors declare that they have no competing interests.

## Authors’ contributions

AMZ designed the study and carried out the protocol and drafted the manuscript. GM partecipated in the design of the study. AR participated in the reclutation of patients. CC performed the statistical analysis. MC participated in its design and coordination. ST partecipated in the design of the study, in the coordination and in the drafting of the study; she gave the contribution to this research as senior author. MD conceived the study and designed the protocol; she gave the contribution to this research as senior author. All authors read and approved the final manuscript.

## Pre-publication history

The pre-publication history for this paper can be accessed here:

http://www.biomedcentral.com/1471-2431/12/175/prepub
